# Prediction of influenza outbreaks in Fuzhou, China: comparative analysis of forecasting models

**DOI:** 10.1186/s12889-024-18583-x

**Published:** 2024-05-25

**Authors:** Qingquan Chen, Xiaoyan Zheng, Huanhuan Shi, Quan Zhou, Haiping Hu, Mengcai Sun, Youqiong Xu, Xiaoyang Zhang

**Affiliations:** 1 The Affiliated Fuzhou Center for Disease Control and Prevention of Fujian Medical University, Fuzhou, 350005 China; 2 The School of Public Health, Fujian Medical University, Fuzhou, 350108 China

**Keywords:** Influenza, XGBoost, SARIMA, Prophet, Holt-Winters, Time series, Prediction model

## Abstract

**Background:**

Influenza is a highly contagious respiratory disease that presents a significant challenge to public health globally. Therefore, effective influenza prediction and prevention are crucial for the timely allocation of resources, the development of vaccine strategies, and the implementation of targeted public health interventions.

**Method:**

In this study, we utilized historical influenza case data from January 2013 to December 2021 in Fuzhou to develop four regression prediction models: SARIMA, Prophet, Holt-Winters, and XGBoost models. Their predicted performance was assessed by using influenza data from the period from January 2022 to December 2022 in Fuzhou. These models were used for fitting and prediction analysis. The evaluation metrics, including Mean Squared Error (MSE), Root Mean Squared Error (RMSE), and Mean Absolute Error (MAE), were employed to compare the performance of these models.

**Results:**

The results indicate that the epidemic of influenza in Fuzhou exhibits a distinct seasonal and cyclical pattern. The influenza cases data displayed a noticeable upward trend and significant fluctuations. In our study, we employed SARIMA, Prophet, Holt-Winters, and XGBoost models to predict influenza outbreaks in Fuzhou. Among these models, the XGBoost model demonstrated the best performance on both the training and test sets, yielding the lowest values for MSE, RMSE, and MAE among the four models.

**Conclusion:**

The utilization of the XGBoost model significantly enhances the prediction accuracy of influenza in Fuzhou. This study makes a valuable contribution to the field of influenza prediction and provides substantial support for future influenza response efforts.

## Introduction

Influenza, a highly contagious respiratory disease, presents a significant global public health challenge [1]. Annual influenza outbreaks not only place a tremendous strain on healthcare systems, resulting in economic losses, but in extreme cases, can also lead to mass casualties [2]. Effective influenza forecasting and prediction are, therefore, of paramount importance to facilitate the timely allocation of resources, the development of vaccination strategies, and the implementation of targeted public health interventions [3, 4]. These measures play a crucial role in mitigating the spread of influenza, enhancing public health protection, and minimizing adverse social and economic consequences [4]. Time series analysis has emerged as a pivotal tool for examining trends in influenza pandemics and forecasting, as it enables the capture of seasonal patterns and fluctuations in influenza cases [5].

As the capital city of Fujian Province in China, Fuzhou is also deeply affected by influenza outbreaks. An in-depth comprehension of the dynamics of influenza transmission in Fuzhou and the creation of precise prediction models hold significant importance in public health planning and influenza response [6]. In recent years, the availability of historical influenza data has increased, data analysis techniques have advanced, and the development of advanced modeling techniques has opened new avenues for enhancing the precision and reliability of influenza forecasts [7, 8]. These advancements offer fresh opportunities to achieve a more comprehensive and accurate grasp of influenza transmission patterns within Fuzhou, as well as to make precise predictions regarding the scale and timing of influenza outbreaks.

Our study seeks to make a meaningful contribution to the field of influenza prediction by creating and assessing several models designed to forecast influenza outbreaks. We employed a variety of prediction models, encompassing the Seasonal Autoregressive Integrated Moving Average Model (SARIMA), Prophet, Holt-Winters, and Extreme Gradient Boosting (XGBoost) models. Each of these models possesses distinctive strengths and has exhibited their effectiveness across various forecasting scenarios.

The SARIMA model employs time series analysis techniques to capture seasonal and temporal patterns in influenza data, incorporating considerations for seasonality, trends, and lag effects [9]. The Prophet model, developed by Facebook, is designed for time series data with both seasonality and holiday effects [10]. In contrast, the Holt-Winters model is dedicated to accounting for the seasonality and trend components within the data [11]. On the other hand, the XGBoost model leverages the capabilities of machine learning and integration techniques to enhance the accuracy of influenza prediction [12, 13]. Therefore, based on previous research support and considering the seasonal characteristics and complex trends of influenza data in Fuzhou, we selected the four prediction models mentioned above. We also acknowledge that this choice may introduce potential biases. We attempted other prediction models, such as Support Vector Machine (SVM) [14], Long Short-Term Memory (LSTM) [5] and random forest (RF) [15], but the preliminary experimental results did not meet our expectations. This may be because these models are more suitable for data types that include other predictive variables.

In this study, we have conducted a comparative analysis of the performance of four models, resulting in the development of an influenza prediction model that is highly accurate and dependable, suitable for Fuzhou. Our research delves into the application of various models to time series data, with the overarching goal of identifying the optimal influenza prediction model that will contribute to the protection of public health.

## Methods

### Data sources

In 1957, the Chinese National Influenza Center (CNIC) was established in China, followed by the Chinese Influenza Surveillance Network (CISN) [16]. Under the guidance of CNIC, the network covers laboratories and medical institutions throughout the country, forming an extensive and intensive monitoring network. We acquired monthly influenza cases data for Fuzhou City from the CISN. The dataset was exported by professionals from the Fuzhou Center for Disease Control and Prevention. This dataset spanning from January 2013 to December 2022 was partitioned into two distinct subsets: a training set encompassing the period from January 2013 to December 2021 and a test set covering the period from January 2022 to December 2022. Meanwhile, our dataset only includes monthly counts of reported influenza cases and does not contain any outliers or missing values. Models were developed using the training data and assessed for their performance on the test set. Subsequently, the model was evaluated using both the training and test sets.

Additionally, we employed pipelining techniques to decouple the data preprocessing steps from the model training process, ensuring that test data information is not leaked during data processing. Pipelining data processing is a technique that separates and integrates data preprocessing steps with model training steps [17]. Its primary objective is to ensure that test data information is not leaked during the data processing process, thereby avoiding biases in model evaluation results caused by data leakage.

### Seasonal Autoregressive Integrated Moving Average Model (SARIMA)

The SARIMA is a model that combines seasonal differencing and ARIMA model to effectively model time series data with cyclical patterns [18]. The SARIMA model is based on stationary time series data, so it is necessary to determine whether the data is stationary before modeling. There are two main methods for testing the stationarity of a time series. The first method is the graphical method, which involves observing the time series plot or the autocorrelation function (ACF) and partial autocorrelation function (PACF) diagrams [19]. ACF and PACF are commonly used to identify patterns in data, such as whether it is suitable to use an Autoregressive (AR) model or Moving Average (MA) model. The second method is the unit root test, with the Augmented Dickey-Fuller (ADF) test being a commonly used method [20]. If the *p*-value of the ADF test is less than 0.05, the series can be considered stationary. Otherwise, differencing or logarithmic transformation is needed to convert the non-stationary series into a stationary one. By applying these two methods to the influenza case data in Fuzhou from 2013 to 2019, we can analyze whether the data is stationary.

The SARIMA(p, d, q) (P, D, Q)_s_ comprises a total of seven parameters, which can be categorized into two groups: three non-seasonal parameters (p, d, q) and four seasonal parameters (P, D, Q)_s_. Specifically, p denotes the autoregressive order of the trend, d stands for the differential order of the trend, q represents the moving average order of the trend, P is the seasonal autoregressive order, D signifies the seasonal differential order, Q denotes the seasonal moving average order, and s indicates the number of time steps within a single seasonal cycle. The general expression for SARIMA is Eq. (1).

1 $${\phi }_{p}\left(B\right){\widetilde{\phi }}_{p}\left({B}^{s}\right){y}_{t}^{*}={\theta }_{q}\left(B\right){\widetilde{\theta }}_{Q}\left({B}^{s}\right){\varepsilon }_{t}$$

In Eq. (1), ${y}_{t}^{*}=A\left(t\right)+{\Delta }^{d}{\Delta }_{s}^{D}{y}_{t}={\left(1-B\right)}^{d}{\left(1-{B}^{s}\right)}^{D}{y}_{t}$, ${\phi }_{p}\left(B\right)$ represents a non-seasonal autoregressive lag polynomial, ${\widetilde{\phi }}_{p}\left({B}^{s}\right)$ represents a seasonal autoregressive lag polynomial, ${\theta }_{q}\left(B\right)$ represents a non-seasonal moving average lag polynomial, ${\widetilde{\theta }}_{Q}\left({B}^{s}\right)$ represents a seasonal moving average lag polynomial, $A\left(t\right)$ represents a trend polynomial, and can be constant. Finally, we need to apply the Ljung-Box method to perform a goodness-of-fit test on the model [21]. The purpose is to analyze the autocorrelation of the residual sequence. If the p-value is less than 0.05, it indicates that the model's fit is not good. If the p-value is greater than 0.05, it indicates that the model's fit is good.

### Prophet model

The Prophet model, developed by the Facebook team in 2017, is a powerful tool for time series data forecasting [22]. It can handle time series data with both linear and non-linear growth, as well as multiple seasonality patterns. Prophet is known for its ease of use, speed, and automatic prediction of future trends [23]. The automatic here means that Prophet automatically identifies patterns and trends in the data and generates accurate predictions. It excels not only in handling time series data with outliers but also in dealing with missing values.

The Prophet model decomposes the input time series into three components: trend, seasonality, and holiday effects. The basic formula for the Prophet model is represented in Eq. (2).

2 $$y\left(t\right)=g\left(t\right)+s\left(t\right)+h\left(t\right)+{\epsilon }_{\left(t\right)}$$

where $g\left(t\right)$ represents the overall trend and does not include any cyclical factors, such as long-term growth or decline. It is the core term of the Prophet model, used to fit the non-periodic changes in the time series. Its expression is shown in Eq. (3).

3 $$g(t)=\frac{C}{1+{e}^{(-k(t-b))}}$$

In Eq. (3), $C$ represents capacity; $k$ represents the growth rate of the model; $b$ represents the model offset. When $t$ increases, $1+{e}^{(-k(t-b))}$ approaches to 1, or $g(t)$ approaches to $C$.

The $s(t)$ indicates cyclical factors, and the period factor of this term adopts the Fourier series, and the expression is shown in Eq. (4).

4 $$s(t)=\sum_{n=1}^{N}({a}_{n}\mathit{cos}(\frac{2\pi nt}{T})+{b}_{n}\mathit{sin}(\frac{2\pi nt}{T}))$$

In Eq. (4), $T$ represents cycles; $n$ represents half the number of cycles used in the model.

The $h(t)$ represents repeated but non-cyclical factors, such as holidays. This will separate the factor affecting the festival. The expression is shown in Eq. (5):

5 $$h\left(t\right)=Z\left(t\right){\kappa }_{i}, \kappa \sim Normal(0,{v}^{2})$$

In Eq. (5), Where each festival is represented by $i$; ${D}_{i}$ is a collection of festivals; $Z(t)=[1(t\in {D}_{i}),\cdots ,1(t\in {D}_{i})];1(t\in {D}_{i})$ is an indicator function, if $t$ is a function of the number of festivals in ${D}_{i}$ in the set, the value of $1(t\in {D}_{i})$ is 1; if $t$ is in ${D}_{i}$, the value of $1(t\in {D}_{i})$ is 0; ${\kappa }_{i}$ is the parameter of each festival, representing the effect on each festival.

And the final ${\epsilon }_{\left(t\right)}$ represents the measurement error.

### Holt-Winters model

The Holt-Winters model is a widely-used method for time series analysis and forecasting [24]. This method extends the Holt model by introducing a Winters period term, also known as the seasonal term. The Winters term is particularly valuable when dealing with time series data that exhibit fluctuating behavior at fixed time intervals, such as monthly, quarterly, or weekly data. One of the key strengths of the Holt-Winters model is its applicability to non-stationary time series data that contain linear trends and cyclical fluctuations. It achieves this by utilizing the Exponential Smoothing Method (EMA), which enables the model parameters to continuously adapt to the changes in the non-stationary series. This adaptive nature allows the model to provide short-term forecasts of future trends effectively.

The Holt-Winters model can be categorized into additive and multiplicative models. The choice between these two models depends on the nature of the seasonal variations within the time series data. Additive models are typically chosen when the seasonal variations exhibit a roughly constant pattern over the time series, while multiplicative models are preferred when the seasonal variations vary proportionally with the level of the time series.

### Extreme Gradient Boosting (XGBoost) model

XGBoost is an implementation of the gradient boosting integration method used for solving classification and regression problems [25]. It operates as a tree-based model, allowing the stacking of any number of trees. Each additional tree is designed to minimize the error, collectively working towards creating a strong predictor. The fundamental concept behind XGBoost is to amalgamate numerous simple and weak predictors to form a robust and accurate predictor.

In this study, we employed the grid-search method to find the optimal parameter combination for the XGBoost model to address the problem. Grid-search is an exhaustive search method that traverses the specified parameter space and evaluates the performance of each parameter combination to find the best one [26]. We defined a set of parameters to be optimized, including Nrounds, SubSampRate, ColSampRate, Depth, MinChild, and eta. To evaluate the performance of each parameter combination, we used five-fold cross-validation. By utilizing five-fold cross-validation, we can comprehensively evaluate the performance of each parameter combination and avoid overfitting to a specific dataset [27]. Additionally, we can systematically search the parameter space using the grid-search method to find the optimal parameter combination and optimize the performance of the XGBoost model.

XGBoost calculates predictions based on Eq. (6) and Eq. (7).

6 $${\widehat{y}}_{i}=\sum_{k=1}^{K}{f}_{k}\left({x}_{i}\right),{f}_{k}\in \mathcal{F}$$

7 $${f}_{t}\left(x\right)={w}_{q\left(x\right)},w\in {R}^{T},q:{R}^{d}\to \{1,2,\cdots ,T\}$$

where ${\widehat{y}}_{i}$ represents the prediction, ${x}_{i}$ represents the feature vector, ${f}_{k}\left({x}_{i}\right)$ represents the value computed for each tree, and $K$ represents the total number of trees. $q\left(x\right)$ represents a function that assigns the feature $x$ attribute to a specific leaf of the current tree $t$. ${w}_{q\left(x\right)}$ represents then the leaf score of the current tree $t$ and the current feature $x$. When the model is trained, XGBoost prediction can be boiled down to identifying the leaves of each tree based on the features and summing the values of each leaf.

### Model selection

We evaluated the performance of the four models using three common evaluation metrics for linear regression models: Mean Squared Error (MSE), Root Mean Squared Error (RMSE), and Mean Absolute Error (MAE).

MSE is a widely used metric for quantifying the disparity between a model's predicted values and the actual observed values, serving as an indicator of how well the model fits the provided dataset. MSE is calculated by finding the mean of the squared differences between the predicted values and the actual observed values.

8 $${\text{MSE}}=\frac{1}{n}\sum_{i=1}^{n}{\left({\widehat{y}}_{i}-{y}_{i}\right)}^{2}$$

RMSE is another commonly employed metric to assess the dissimilarity between a model's predicted values and the actual observed values, providing insight into the model's fit to the given data. RMSE is determined by computing the mean of the squared differences between predicted values and actual observations, followed by taking the square root of the result.

9 $${\text{RMSE}}=\sqrt{\frac{1}{n}\sum_{i=1}^{n}{\left({\widehat{y}}_{i}-{y}_{i}\right)}^{2}}$$

In contrast, MAE is also a frequently used measure for assessing the divergence between a model's predicted and actual observations, indicating the model's fit to the provided data. MAE is derived by calculating the mean of the absolute differences between the predicted and actual observations.

10 $${\text{MAE}}=\frac{1}{n}\sum_{i=1}^{n}\left|{\widehat{y}}_{i}-{y}_{i}\right|$$

Smaller values of MSE, RMSE, and MAE indicate a better fit of the model.

### Statistical analysis

The data processing and modeling for this study were conducted using R software (version 4.2.1, The R Foundation). We developed various models primarily utilizing packages such as forecast, prophet, and xgboost. Additionally, we employed the ggplot2 package to create visual representations of the results through graphs and charts. The significance level was predetermined at 0.05.

### Ethical approval

This study was approved by the Ethical Review Committee of the Fuzhou Center for Disease Control and Prevention (Approval No. IRB2020008).

## Results

### Characteristics of influenza cases

**Fig. 1:**
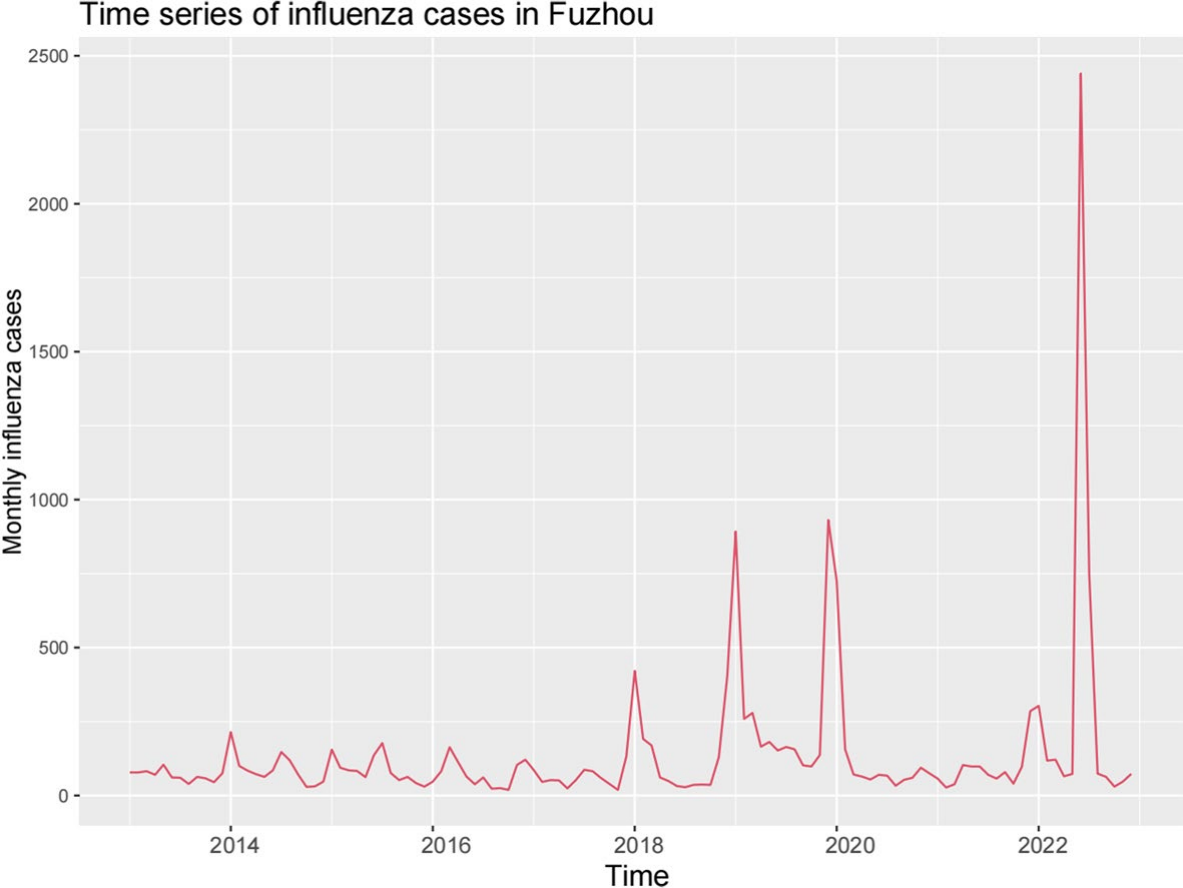
Time series of monthly influenza cases in Fuzhou from January 2013 to December 2022

Between January 2013 and December 2022, a total of 16,355 cases of influenza were reported in Fuzhou, with the highest number of reported cases reaching 2,440 in June 2022. The time series chart reveals that the peak incidence of influenza in Fuzhou predominantly occurs from December to February of the following year, indicating a pronounced high-incidence pattern during the winter and spring months. In general, the influenza cases data demonstrates a notable increasing trend with significant fluctuations (refer to Fig. 1).

**Fig. 2:**
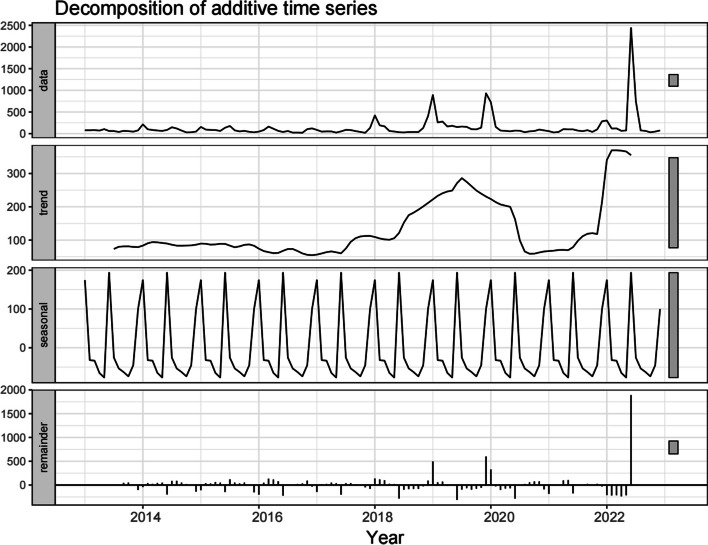
The monthly influenza cases data in Fuzhou were decomposed into trend part, seasonal part and random part

The Augmented Dickey-Fuller (ADF) test confirmed the stability of the influenza data in this study (*p* < 0.01). Additionally, we decomposed the influenza data into its trend, seasonal, and random components, revealing a distinct seasonal incidence pattern of influenza in Fuzhou (see Fig. 2).

### Forecasting the cases of influenza by the SARIMA model

**Fig. 3:**
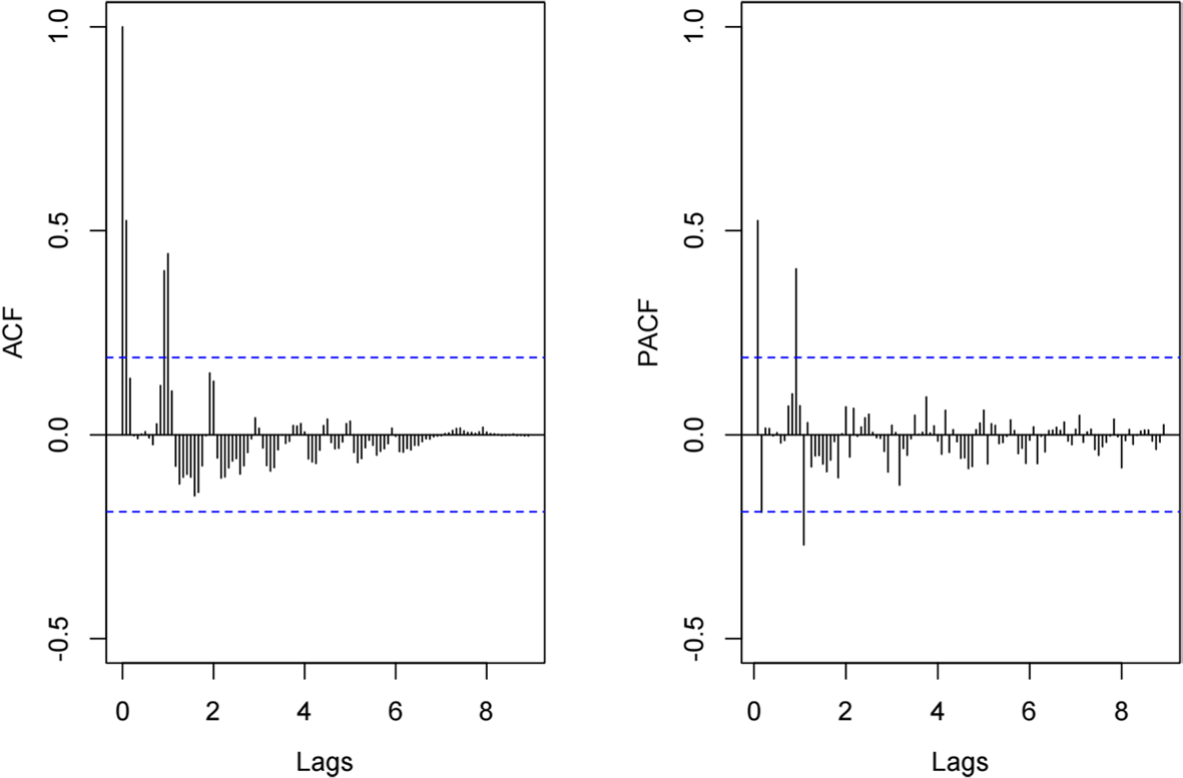
(**A**) Autocorrelation function (ACF) and (**B**) partial autocorrelation function (PACF) diagrams for monthly cases of influenza in Fuzhou

**Fig. 4:**
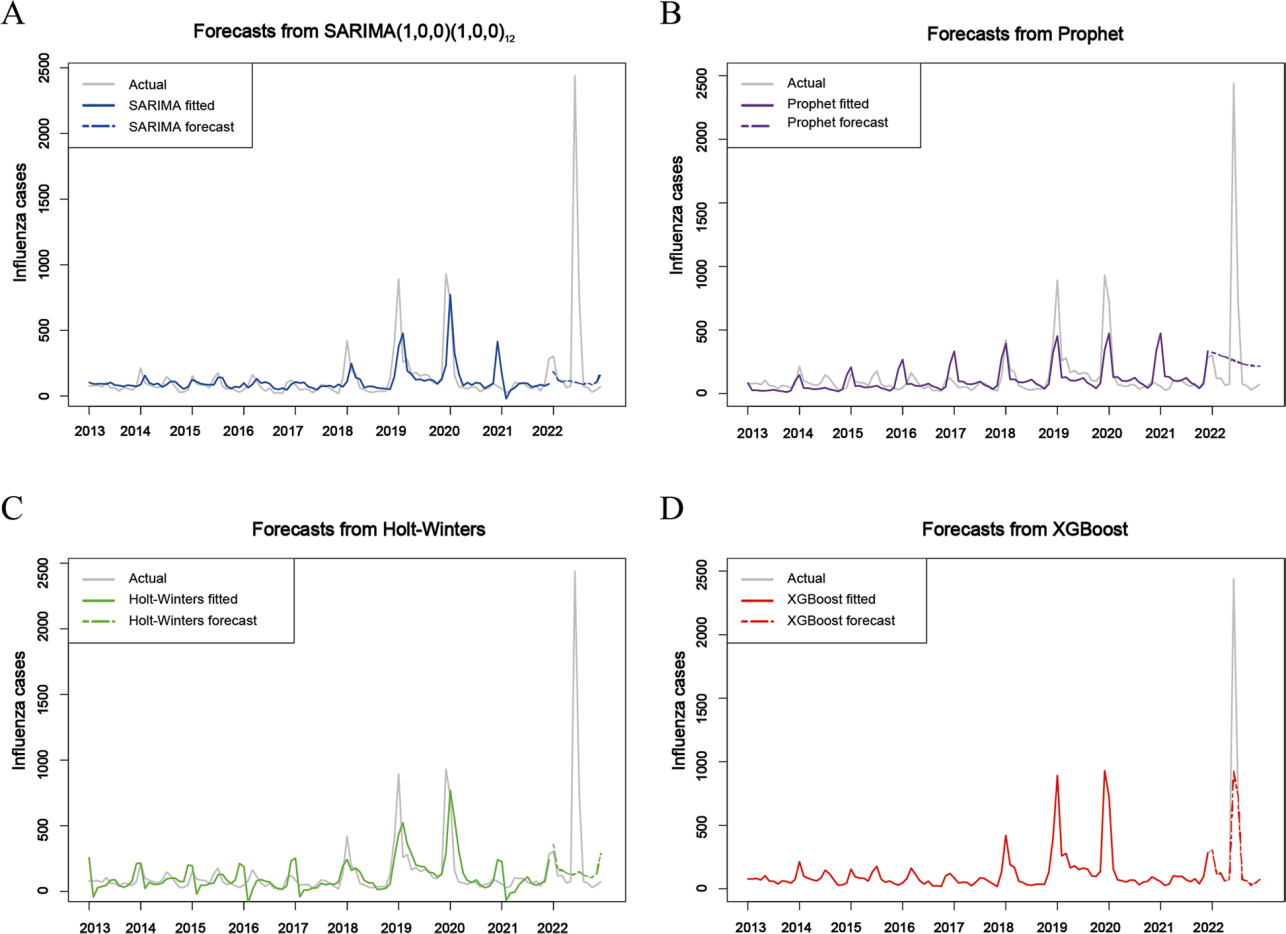
The fitting and prediction performance of four models in this study. (**A**) SARIMA(1, 0, 0) (1, 0, 0)12 model, (**B**) Prophet model, (**C**) Holt-Winters model, (**D**) XGBoost model

First, the ADF test supports the stationarity of the data (t = -4.2109, *p* < 0.01). As a result, both the parameters d and D of the SARIMA model are set to 0. Additionally, based on the insights from Fig. 2, we deduce that the parameter s of the SARIMA model should be 12. Subsequently, we determined that the values of the remaining parameters p, q, P, and Q should be either 0 or 1 through the examination of the ACF and PACF plots (refer to Fig. 3). Finally, utilizing the autoarima function, we identified the optimal SARIMA model with the lowest AICc value. The optimal SARIMA model is SARIMA(1, 0, 0) (1, 0, 0)_12_, with the minimum AIC, AICc, and BIC values of 1332.460, 1332.850, and 343.190 (Table 1), respectively. The residual sequence of the SARIMA(1, 0, 0) (1, 0, 0)_12_ model exhibits characteristics of white noise (*p* = 0.513). The SARIMA(1, 0, 0) (1, 0, 0)_12_ model demonstrated excellent performance in fitting and predicting influenza cases data. The fitted MSE, MAE and RMSE were calculated as 12,145.197, 55.406, and 110.205, respectively (Table 2). The performance of the SARIMA(1, 0, 0) (1, 0, 0)_12_ model is visually presented in Fig. 4A.

**Table 1:** Parameters of the SARIMA(1, 0, 0) (1, 0, 0)_12_ model

		**ma1**	**sma1**
**Coefficients**		0.509	0.388
	**SE**	0.083	0.083
**AIC**		1332.460	
**AICc**		1332.850	
**BIC**		343.190

**Table 2:** Performance of the SARIMA(1, 0, 0) (1, 0, 0)_12_, Prophet, Holt-Winters and XGBoost models

Model	Training set			Test set		
	**MSE**	**MAE**	**RMSE**	**MSE**	**MAE**	**RMSE**
**SARIMA**	12,145.197 (1741.00- 22,844.000)	55.406 (37.910–73.400)	110.205 (65.000–159.200)	491,525.213 (359,788.000–1368784.000)	290.543 (-73.100–658.100)	701.089 (80.500–1481.500)
**Prophet**	11,827.128 (4026.000–20164.000)	65.025 (48.060–81.830)	108.753 (72.000–148.000)	441,990.518 (-303,495.000–1197761.000)	360.579 (35.700–683.100)	664.824 (136.700–1335.400)
**Holt-Winters**	13,159.930 (4609.000–21985.000)	67.574 (50.080–85.390)	114.717 (78.500–153.900)	481,020.478 (-348,238.000–1353383.000)	303.832 (-86.100–649.200)	693.556 (106.800–1476.000)
**XGBoost**	0.007 (0.003–0.012)	0.060 (0.048–0.071)	0.087 (0.061–0.114)	189,937.080 (-156,269.000–543361.000)	128.686 (-107.400–368.800)	435.818 (4.900–1052.300)

### Forecasting the cases of influenza by the Prophet model

**Fig. 5:**
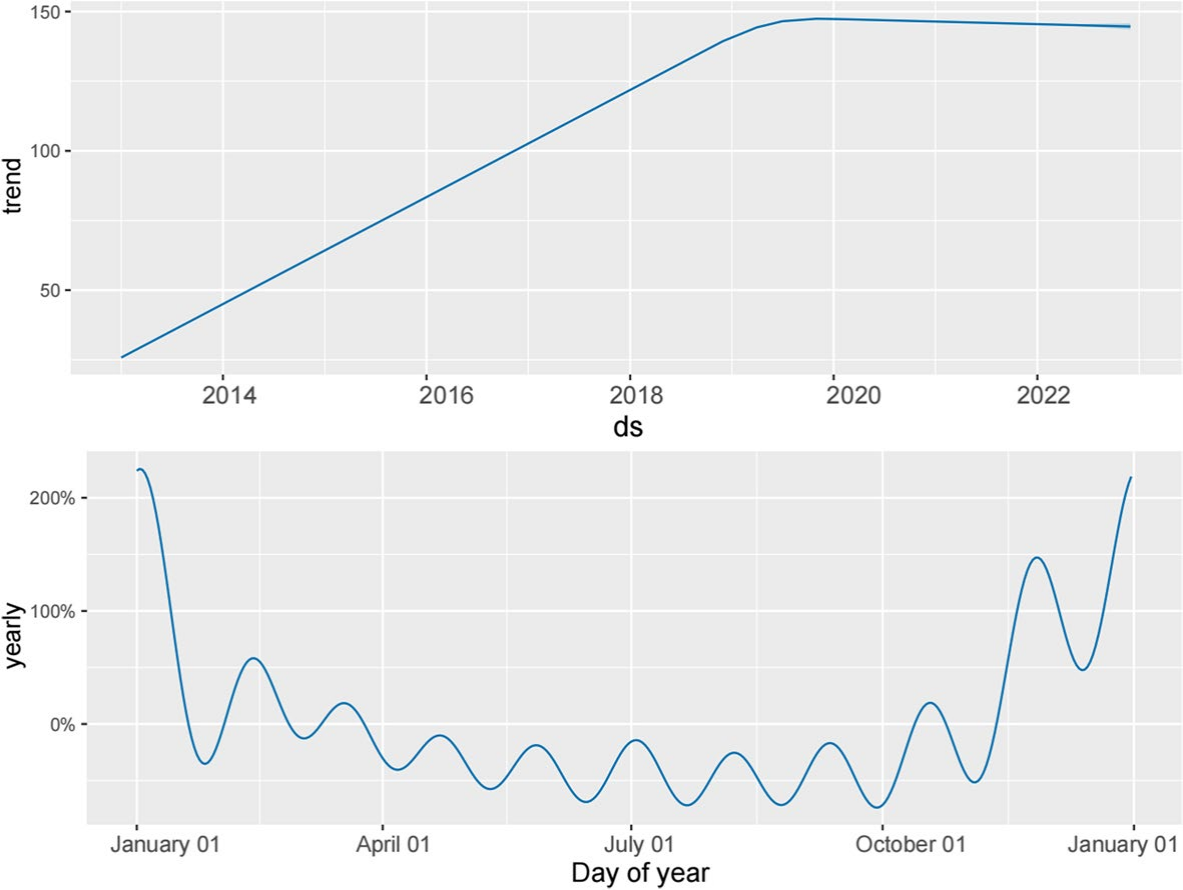
Analysis of each component of the Prophet model

Given the observed increasing trend and annual seasonality in influenza cases in Fuzhou (refer to Fig. 5), we configured the growth parameter as linear and set the annual seasonality parameter to TRUE. In addition, the interval_width parameter of the Prophet model is set to 0.95, the periods parameter is set to 12, and the freq parameter is set to MS. Upon conducting a fitting analysis on the training dataset using the Prophet model, we obtained the following results: the fitted MSE, MAE, and RMSE were calculated as 11,827.128, 65.025, and 108.753, respectively (Table 2). The performance of the Prophet model is visually presented in Fig. 4B.

### Forecasting the cases of influenza by the Holt-Winters model

The time series data for monthly influenza cases in Fuzhou exhibits noticeable seasonal fluctuations. We constructed both Holt-Winters additive and multiplicative models using the training dataset. The model with the smallest sum of squared residuals (SSE) and RMSE was chosen as the optimal model, and the model's smoothing parameters were automatically determined. After a comprehensive comparison, the Holt-Winters additive model emerged as the preferred choice for exponential smoothing. The training dataset was analyzed using the Holt-Winters additive model, resulting in fitted values of MSE, MAE, and RMSE at 13,159.930, 67.574, and 114.717 for the respective components (Table 2). The performance of the Holt-Winters additive model is illustrated in Fig. 4C. 

### Forecasting the cases of influenza by the XGBoost model

The selection of appropriate hyperparameters is of paramount importance when utilizing the XGBoost model. We employed grid-search and five-fold cross-validation to identify the optimal combination of hyperparameters, which included Nrounds = 300, SubSampRate = 0.7, ColSampRate = 0.4, Depth = 7, MinChild = 2, and eta = 0.07. Subsequently, we employed the optimal XGBoost model to train on the training dataset, resulting in fitted values of MSE, MAE, and RMSE at 0.007, 0.060, and 0.087, respectively (Table 2). The performance of the optimized XGBoost model is visualized in Fig. 4D.

### Models comparison

We applied the optimal models of SARIMA, Prophet, Holt-Winters, and XGBoost to forecast the influenza data for Fuzhou in 2022. To assess the models' performance, we compared the actual values from the training set to the model's fitted values, evaluating their fitting performance. Subsequently, we compared the actual values from the test set to the models' predicted values to evaluate their forecasting performance. For the evaluation of both fitting and prediction performance, we utilized three performance metrics: MSE, MAE, and RMSE. Smaller values of these metrics indicate superior model performance.

As presented in Table 2, among the four models constructed in this study, the Holt-Winters model exhibited the lowest fitting performance, with a MSE of 13,159.930, MAE of 67.574, and RMSE of 114.717. Conversely, the XGBoost model demonstrated the highest fitting performance, with a MSE of 0.007, MAE of 0.060, and RMSE of 0.087. This indicates that the Holt-Winters model had the least accurate fit to the training data, while the XGBoost model provided the most accurate fit. When it comes to predictive performance, the SARIMA model underperforms with a MSE of 491,525.213, a MAE of 290.543, and an RMSE of 701.089, making it the least accurate in forecasting. Conversely, the XGBoost model shines in predictive performance, boasting a MSE of 189,937.080, a MAE of 128.686, and an RMSE of 435.818, which is notably superior to both the Holt-Winters and Prophet models. These results strongly indicate that the XGBoost model outperforms the other models in terms of fitting and predicting influenza in Fuzhou.

## Discussion

### Major research findings

Our study reveals that influenza epidemics in Fuzhou exhibit a pronounced seasonal and cyclical pattern, with the peak cases predominantly occurring between December and February each year, indicative of a distinct high-incidence pattern during the winter and spring seasons. In general, the influenza cases data in Fuzhou exhibit a clear upward trend with significant fluctuations. Especially during the period from 2018 to 2020, the overall increase in influenza cases in Fuzhou City is closely associated with the outbreak of H1N1 influenza in 2018 and the seasonal H3N2 influenza epidemic in 2019 [28]. Additionally, the continuous increase in population density and the thriving social activities in Fuzhou also contribute to the occurrence of influenza outbreaks. Following the outbreak of COVID-19, Fuzhou implemented strengthened epidemic prevention and control measures in key public places such as schools, leading to effective control of influenza outbreaks in 2020 and 2021 [29]. Time series analysis serves to elucidate the temporal influenza distribution pattern in Fuzhou, aiding in the timely implementation of preventive and control measures, which is crucial for effectively averting outbreaks and epidemics of influenza [30].

The development of influenza prediction models serves as a pivotal scientific foundation for the formulation of strategies to prevent and control influenza epidemics [31]. In this study, we have developed four prediction models, including SARIMA, Prophet, Holt-Winters, and XGBoost models. Leveraging historical monthly influenza cases data from 2013 to 2021, we forecasted future one year's influenza data for Fuzhou. We evaluated the fitting and prediction performance of these models using three metrics.

Firstly, we employed the SARIMA model for influenza forecasting. The SARIMA model is a widely-used time series model that effectively captures the trend and seasonality of data. It has been successfully applied in various infectious disease forecasting studies, including tuberculosis [32], COVID-19 [33], and hemorrhagic fever [34]. By automatically identifying and adjusting the parameters based on the AICc minimum rule, we obtained the optimal SARIMA model, specifically SARIMA(1, 0, 0) (1, 0, 0)_12_ model. However, it is important to note that while the SARIMA model better captures the temporal characteristics of infectious diseases, it requires stable inputs or stable time series data after differentiation, and is unable to predict infectious diseases with nonlinear transmission rates accurately [35]. Subsequently, we explored the use of the Prophet model for influenza prediction. In comparison to the SARIMA model, the Prophet model exhibited superior performance in fitting and predicting influenza cases in Fuzhou. This can be attributed to the Prophet model's ability to automatically detect and adapt to seasonality, trend, and holiday effects present in the data. It is important to mention that Xie C et al. have successfully applied the Prophet model to predict the daily reported incidence of hand, foot and mouth disease in Hubei [36]. Next, we also used the Holt-Winters model for influenza prediction. In this study, the fitting performance of the Holt-Winters additive model was lower than that of the SARIMA model, but its prediction accuracy was higher than that of the SARIMA model. This may be due to the fact that the Holt-Winters model is given different weights in size according to the proximity of the data, and the recent data have a greater impact on the results, while the distant data have a smaller impact, which is suitable for analysing data that do not change much over time. Many scholars have widely applied the Holt-Winters model to the prediction of infectious diseases, such as acute haemorrhagic conjunctivitis [37], dengue fever [38] and COVID-19 [39].

The previously mentioned three models are primarily designed for fitting and predicting linear data. However, the influenza cases data in Fuzhou exhibits a non-linear trend and is influenced by factors such as COVID-19, population movement, and climatic conditions. As a result, the performance of these three models in terms of fitting and prediction is not satisfactory. Machine learning methods such as Support Vector Regression (SVR) [40] and XGBoost [41] can be employed to address this limitation. These approaches have proven to be effective in handling non-linear infectious disease data and can achieve higher prediction accuracy.

XGBoost is an integrated decision tree-based learning model with powerful predictive capabilities [41]. It has a larger delayed pruning penalty compared to traditional gradient boosting decision trees, which makes the model less prone to overfitting [42]. Our study fills the gap in the use of XGBoost models for predicting time series data of influenza cases and provides a more accurate method for predicting influenza cases in Fuzhou. The XGBoost model has demonstrated the potential to predict sudden, widespread influenza outbreaks during the winter of 2022. This may be because the XGBoost model utilizes an ensemble learning framework that combines multiple decision trees to make predictions. This approach allows the model to learn from the strengths of individual decision trees and reduce biases, resulting in improved prediction accuracy. Simultaneously, We employed extensive feature engineering techniques to derive informative features from the raw data. We also conducted feature selection to identify the most relevant variables for the prediction task. This process helped in capturing the key indicators and patterns associated with influenza outbreaks, enhancing the model's predictive power.

By utilizing historical influenza case data as features and influenza data from a future period as target variables, we trained the XGBoost model. This model incorporates machine learning and integration techniques to better capture complex relationships and patterns in influenza data, thereby improving the accuracy of predictions. Additionally, the XGBoost model exhibits high flexibility and can adapt to different data characteristics and prediction requirements [43]. It automatically handles missing values, outliers, and non-linear relationships, thereby further improving prediction accuracy. Evaluation of the model's performance using metrics such as MSE, MAE, and RMSE reveals that the XGBoost model demonstrates superior fitting and prediction performance for influenza cases in Fuzhou.

We utilized several influenza prediction models in our study and conducted a comparative evaluation. Each model possesses unique strengths and is suited to different scenarios. The XGBoost model excels in feature engineering and performance, the SARIMA model is suitable for capturing trends and seasonality in the data, the Prophet model automatically adapts to the data's characteristics, and the Holt-Winters model is well-suited for analyzing seasonal data. In future research, it would be beneficial to explore the combination and optimization of these models to enhance the accuracy and effectiveness of influenza prediction. When developing influenza prediction models, it is crucial to consider the impact of factors such as preventive and control measures during COVID-19 [44, 45] and the climate environment [46, 47].

Influenza remains a significant respiratory infectious disease globally, exerting a profound influence on public health and the economy. The XGBoost model developed in our study demonstrated excellent performance in predicting influenza in Fuzhou, providing accurate predictive information to the public health sector. This information can aid in the development of effective interventions to protect population health and ensure societal stability.

### Limitations

There are several limitations to this study. Firstly, the models were tested using data specifically from Fuzhou, and it is unclear how applicable they would be to other regions or diseases without adjustments. Secondly, the complexity of the XGBoost model may present challenges in understanding and interpreting the model for public health officials without technical expertise. This could limit its practical utility in real-world settings. Thirdly, we did not account for the potential influence of external variables such as meteorological factors and air pollutants on the outcomes. We plan to develop relevant predictive models in the future to thoroughly investigate the impact of these factors on the outcomes, thereby enhancing the performance of the predictive models. Finally, our study employed only a single predictive modeling approach without considering the integration of multiple predictive models. This decision was made to promptly deploy the relevant models in practical influenza control efforts. However, future research will focus on integrating various short-term predictive models to improve prediction accuracy and reliability.

## Conclusions

In this study, we have gained a profound understanding of the transmission dynamics of influenza in Fuzhou and have developed an accurate and reliable model for predicting influenza. The epidemic of influenza in Fuzhou exhibits a seasonal and cyclical pattern, with the peak season predominantly occurring during the winter and spring each year, showing a noticeable upward trend. We have developed and compared the performance of four prediction models, including SARIMA, Prophet, Holt-Winters, and XGBoost models. Our findings reveal that the XGBoost model outperformed the others in fitting and predicting influenza cases in Fuzhou.

The application of the XGBoost model holds the potential to assist in the efficient allocation of resources, the formulation of vaccine strategies, and the implementation of targeted public health interventions. This, in turn, can contribute to the mitigation of influenza spread and the reduction of its adverse impacts on public health and the economy. Our study represents a valuable contribution to the field of influenza prediction, offering substantial support for future influenza response efforts.

## Data Availability

The source code and the datasets used in this study are freely available at https://github.com/Xuyqiong/Influenza-Forecasting-Models.
